# Tracking the origin of bacterial DNA in blood: Indication of localized and sporadic transfer from other body sites

**DOI:** 10.1080/29933935.2025.2482771

**Published:** 2025-04-01

**Authors:** Maija Rozenberga, Rihards Saksis, Ilze Elbere, Liga Birzniece, Monta Briviba, Ilze Konrade, Janis Klovins

**Affiliations:** a Latvian Biomedical Research and Study Centre, Translational Omics group, Riga, Latvia; b Department of Internal Diseases, Riga Stradins University, Riga, Latvia

**Keywords:** Blood microbiome, circulating microbiome, circulating microbial DNA, microbiome stability, microbiome origin

## Abstract

Recent studies propose the existence of a blood microbiome, but its composition, origin, and dynamics remain largely unresolved. In this pilot study, we analyzed the bacterial DNA present in the blood of 10 volunteers by comparing the taxonomic profiles of 16S rRNA gene sequences from skin, vaginal, oral, and fecal samples. After applying stringent decontamination protocols, we detected bacterial DNA in all blood samples, predominantly from the *Pseudomonas* genus. A key finding was the identification of 32 unique Amplicon Sequence Variants (ASVs) that were identical between blood and a single body site within individual participants, with no overlap between multiple body sites or across different participants. This participant-specific overlap suggests a true biological origin of bacterial DNA in blood, likely stemming from localized bacterial migration, such as from the skin. Additionally, 27.4% of the ASVs in blood were found in other body sites, with the highest overlap observed in skin samples. Furthermore, 25.3% of blood ASVs persisted after three months, suggesting a consistent pattern in the bacterial DNA composition detected in blood over time. These findings deepen our understanding of the blood microbiome and provide a basis for future research linking blood microbiota to health and disease phenotypes.

## Introduction

Different microbial communities are found almost everywhere in and on the human body. However, commensal microbiome research is mainly focused on key sites such as the gut, skin, mouth, and vagina, and for a long time, human blood has been perceived as a sterile environment. This is because the presence of any microorganisms in blood was historically associated only with infectious diseases. However, new sequencing and culturing approaches have revealed some proof on the presence of microbial signatures also in the blood of healthy individuals.^11,58^

Several studies have suggested that the blood microbiome potentially originates from other locations, particularly in cases of liver and gastrointestinal tract diseases.^15,43,52,53^ Moreover, microbial signatures in the blood microbiome have been suggested as valuable biomarkers for diseases such as hypertension, type 2 diabetes, and cancer, where increased intestinal permeability is observed.^24,44,61^ Nevertheless, the likely source of a healthy human blood microbiome is still unclear. In principle, the blood microbiome is possibly established either by vertical or horizontal transmission (or a combination of both). While one cannot exclude vertical microbial transmission *in utero* that might occur from mother to fetus via placenta or umbilical cord a more realistic hypothesis suggests a potential to acquire the microbiota via horizontal transmission where translocation of bacteria from richer niches, most likely from minor skin or mucosa injuries during daily activities contributes to the formation of blood microbiome.^11,26,42,58^ However, a recent study suggests that although there may be transient and sporadic translocation of microbes into the bloodstream from other body sites there is not enough evidence that detected microbes are permanent residents in the blood environment which would be applicable to the definition of the term “microbiome”.^55^

It is also still unclear whether the genetic material found in blood represents viable, non-viable, or dormant bacteria or only their remains, as current studies provide somewhat controversial results on the viability measures.^11,43,49,60^ Blood is certainly a low microbial biomass microbiome sample, and it is very sensitive to contamination from the environment and laboratory reagents as well as cross-contamination in sample processing procedures. Therefore, special attention should be paid to the inclusion and analysis of negative controls in all sample treatment steps to avoid false-positive results.^11,28,37,50^

The number of studies focusing on the characterization of the blood microbiome is increasing. However, many aspects, including the stability of the potential blood microbiome composition over time remain elusive. In this study, we aimed, for the first time, to analyze the composition, potential origin, and longitudinal dynamics of the microbial signatures present in the blood of a pilot group of volunteers from the general population.

## Methods

### Study design and sample collection

Ten individuals from the general population were enrolled in the study in 2020, meeting the following inclusion criteria: age 18–64 years, no use of antibiotics in the last 2–3 months, no presence of acute gastrointestinal disease, and no diarrhea within seven days prior to participation in the study. Samples and information on all participants were included in the Genome Database of the Latvian Population (LGDB), and recruitment was organized following previously established practices.^46^ All participants involved in this study provided written informed consent before sample collection. The Central Medical Ethics Committee of Latvia (No. 01–29.1/6359) approved the methods and protocols used in the study.

**Figure 1.**
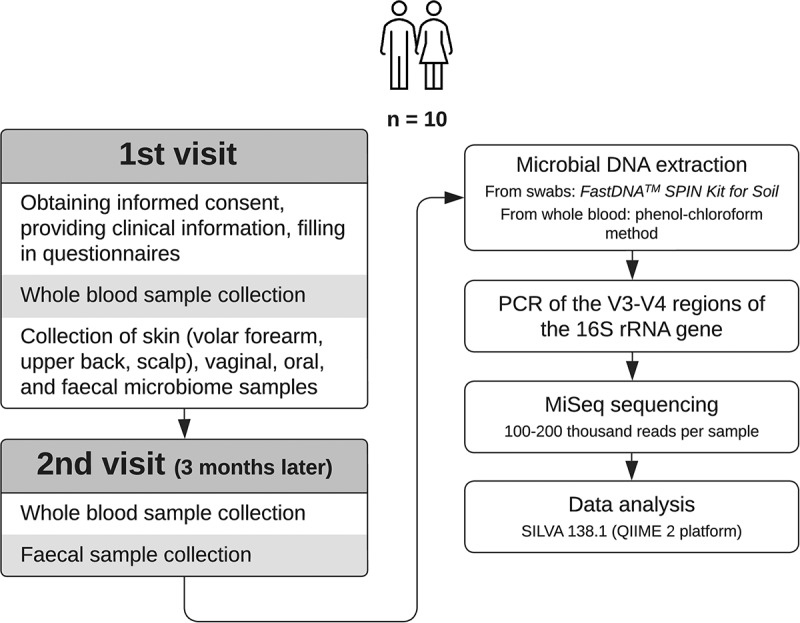
Study design.

Participants were invited to two visits: whole blood samples, swabs from the skin (scalp, upper back, volar forearm), vagina, oral mucosa, and feces were collected during the first visit; while whole blood samples and fecal swabs were collected again three months later (Figure 1). Along with the sample collection, data about the date and time of sample collection, sex, age, height, weight, dietary lifestyle, mode of delivery, smoking and medical history, and medication consumption during the previous two months were collected. Whole blood samples from the median cubital vein were collected by medical personnel after disinfection of the sample collection site; participants were in a fasting state. To avoid microbial DNA contamination from the skin during the blood collection procedure, the blood samples were drawn in three EDTA-containing blood tubes, where either the 2^nd^ or 3^rd^ tube was selected for the subsequent 16S rRNA gene analysis. The samples were stored at + 4°C degrees until further processing. Microbial DNA from blood was isolated within 1–3 days of sample collection.

Oral and skin swabs were collected by medical personnel; vaginal and fecal swabs were collected by participants at home. No food was allowed for 30 minutes before oral microbiome sampling. Vaginal samples from females of reproductive age were not collected during menstruation. eNAT® System with nucleic acid preserving solution (Copan, USA) was used for swab-based collection. Two aliquots were obtained from each sample type. After collection, the samples were stored at room temperature and transported to the laboratory as soon as possible, but no later than within 24 hours, where they were stored at −80°C until the microbial DNA was isolated.

### Sample preparation

Microbial DNA was isolated from the whole blood using the phenol-chloroform method following the standard operation protocol developed by the LGDB.^46^ To reduce contamination, in addition to standard laboratory procedures, sample processing was performed using a surgical face mask and in a laminar flow cabinet.

When all samples from one individual were obtained, microbial DNA from swabs was extracted under sterile conditions in a Biosafety Level 2 cabinet. 200 µl of eNAT® buffer (Copan, USA) containing collected microbial nucleic acids was used to isolate the microbial DNA using FastDNA Spin Kit for Soil (MP Biomedicals, Santa Ana, CA, USA) and FastPrep Instrument following the instructions of the manufacturer. DNA concentrations were evaluated using a Qubit 2.0 fluorometer (Thermo Fisher Scientific, Waltham, MA, USA). Extracted microbial DNA was validated by agarose gel electrophoresis.

Blank controls were included for both extraction methods *‒* phenol-chloroform reagents and the FastDNA Spin Kit for Soil *‒* to ensure they were not contaminated and did not affect the blood microbiome profiles. Blank controls in further steps were treated the same way as biological samples.

### NGS sequencing

The V3-V4 hypervariable region of the bacterial 16S rRNA gene was PCR-amplified using Phusion U Multiplex PCR Master Mix (Thermo Scientific, USA) and a 341F/805 R primer pair. Embedded sample identification sequences were added during a second PCR using appropriate oligonucleotides (see Table S1 for the list of oligonucleotides (Supplementary File 1)). At least one blank control was prepared for every 4–6 samples in the first amplification, and at least one negative control was prepared for every 6–8 samples in the second amplification. Successful amplification of samples and purity of negative controls were determined by agarose gel electrophoresis. The PCR amplicons were purified using NucleoMag magnetic beads (Macherey-Nagel, Düren, Germany), and their quantity and quality were evaluated with the Agilent High Sensitivity DNA kit and Bioanalyzer 2100 instrument (Agilent Technologies, Santa Clara, CA, USA).

Sample sequencing was performed on the Illumina MiSeq platform using the MiSeq Reagent Kit v2 (500-cycles) (Illumina, USA), obtaining at least 100 thousand sequencing reads per sample. All samples from each study participant were placed on the same chip to prevent a batch effect.

Extensive use of negative controls was ensured in all steps (including blank controls when starting sample preparation with a new set of reagents) and working areas. In addition, to ensure sterility, working areas, instruments, and individual reagents were disinfected with UV and disinfectants at all stages, and the work was performed in protective clothing.

### Data analysis

Raw sequencing data were first processed by performing a quality check with FastQC(*v0.11.9*)^4^ and MultiQC(*v1.10*).^18^ Subsequently, data were read into QIIME2(*v2021.2*) software, where most of the remaining analysis was done.

To extract the V3-V4 hypervariable region, the data were trimmed with the QIIME2^8^ Cutadapt plugin^31^ by using the 341F and 805R primer sequences. To check for sequence length and quality after trimming, the Demux plugin was used. Next, data were denoised using the DADA2 plugin,^10^ where the truncation length parameters for forward and reverse reads were set as the second percentile of read length distribution while accounting for the recommended 12 base pair overlap to maximize the number of successfully merged sequences. Data quality trimming was not performed before hypervariable region extraction and denoising to take advantage of DADA2 capabilities of learning from sequencing error rates to make more accurate inference of Amplicon Sequence Variants (ASV). As a result, an ASV table was obtained and further filtered by frequency of five sequences per ASV in at least one sample, to remove possible technical errors in library preparation and sequencing.

To remove possible bacterial contamination of samples during library preparation and sample handling, a frequency-filtered ASV table was exported to R(*v4.1.0*) where the decontam(*v1.12.0*) package^14^ was used in the prevalence mode, where it compares the ASV prevalence (presence/absence) in true samples vs negative control samples. Decontamination and further data processing was done using the phyloseq(*v1.38.0*)^33^ object system. Decontam prevalence mode was chosen due to the fact that for some samples the DNA concentrations were lower than the lowest possible detection limit (<0.05 ng/µl) of the Qubit 2.0 fluorometer (Thermo Fisher Scientific, Waltham, MA, USA). To follow the recommendation by the developers, decontam contaminant classification threshold was determined by the modes from the decontam test statistic P binomial distribution.^23,33^ A list of contaminant ASVs was obtained from all the subgroups and excluded from the filtered ASV table using the QIIME2 feature-table plugin. No manual filtering of contaminant features was performed, as it would introduce a bias regarding the chosen source of contaminant reference as it cannot be 100% comprehensive.

To inspect how sequencing depth affects the Amplicon Sequence Variant (ASV) count and Shannon diversity index values, alpha rarefaction curves were calculated and visualized using the QIIME2 diversity plugin by providing the maximum value of observed features (84545) in a sample from the feature table.

Rarefying or random subsampling without replacement to a depth of 965 ASVs was performed to normalize for the inter-sample library size differences to reliably calculate the various diversity indexes. A rarefying depth of 965 was chosen by examining the alpha rarefaction curves generated in previous steps. Accordingly, to calculate and visualize both phylogenetic and non-phylogenetic diversity metrics on the feature subsample, the diversity plugin was used.

Sequences were then classified using the naïve Bayes classifier by following the RESCRIPt^45^ workflow while using the SILVA(*v138.1*) small subunit non-redundant database with a 99% identity as a reference.^62^ Data from the database were pre-processed to optimize for the highest possible classification confidence. First, only the Archaea, Bacteria, and Eukaryote domains were selected with respective minimal sequence lengths for each domain at 900, 1200, and 1400. Next, the contents of the database were dereplicated to preserve only unique taxa. The V3-V4 region was extracted by providing the 341F and 805R primer sequences. Dereplication was carried out again, and finally, a classifier was trained on the cleaned database.

A bar plot containing each sample and sample group was made to visually assess and compare the taxonomic diversity of said sample groups. Lastly, the phyloseq package was again used to construct additional bar plots for specific sample groups and to construct an NMDS plot with a confidence interval of 95%.

To assess the dynamics of circulating microbial DNA present in the blood over time, the Galaxy website for statistical analysis of biomedical research data was used (https://usegalaxy.org/) where differences between groups were explained using the linear discriminant analysis (LDA) effect size (LEfSe) algorithm with the LefSE analysis tool.^51^ For all tests, the significance threshold = 0.05 and the LDA threshold = 2.0.

## Results

### Characteristics of the study group and samples

Seven female and three male volunteers without acute illness participated in this study. The median age of the study group was 40 (interquartile range (IQR) = 21) years, while body mass index (BMI) was 26 (IQR = 4.3) kg/m^2^. All details about each participant can be found in Table S2 (Supplementary File 1).

The median number of reads obtained per sample was 83,534.50 (IQR = 29,850.75). The depth of sequencing and the used database allowed to classify microorganisms mainly up to the genus level and up to the species level in some cases. A total of 4391 ASVs were detected in all samples, including 4287 ASVs in true samples and 210 ASVs in controls − 28 phyla and 460 genera. After decontamination 4240 ASVs remained in total (28 phyla and 454 genera). To provide reliable results, 11 negative controls were included. After decontamination, these control samples were dominated by bacterial genera such as *Ralstonia* (39.1%), *Hydrogenophilaceae_uncultured* (25.4%), *Undibacterium* (13.7%), *Curvibacter* (5.8%), *Thermus* (4.4%), *Acidovorax* (4.2%), *Lactobacillus* (2.6%), and *Enhydrobacter* (1.3%). The microbial composition of negative controls after decontamination and its frequency and prevalence in true positive sample groups is shown in Figure S1 (Supplementary File 2) and Table S3 (Supplementary File 1), respectively. The most frequent contaminant ASVs were from genera *Ralstonia*, *Prevotella_7, Streptococcus*, and *Undibacterium*. The full list of contaminant taxa at the ASV level is shown in Table S4 (Supplementary File 1).

### Alpha and beta diversity

**Figure 2.**
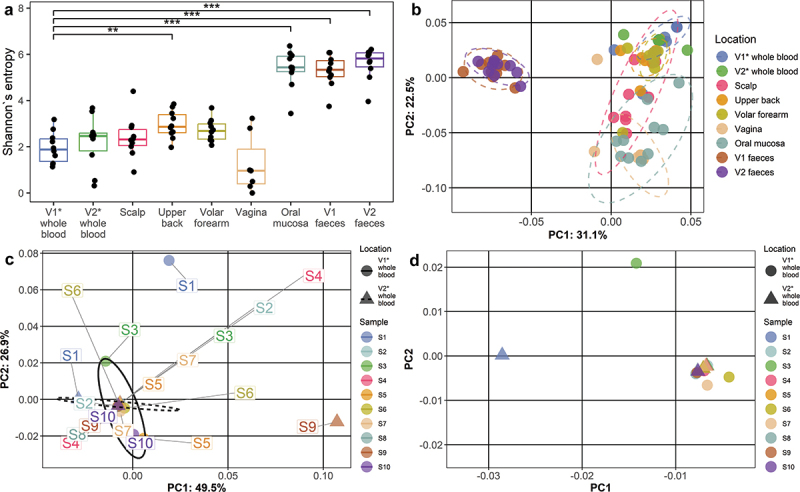
Alpha and beta diversity of microbiome profiles. (a) Boxplots representing alpha diversity Shannon entropy index value distributions for each location. Boxplots depict the median value and interquartile ranges of data in each group. Dots beyond the bounds of the whiskers represent outliers. (b) Representation of beta diversity for all true samples with PCoA weighted UniFrac distance ordination plot. Ellipses represent multivariate normal distribution (CI = 0.95) for each location. (c) Representation of beta diversity only for the first and second visit blood samples with PCoA weighted UniFrac distance ordination plot. Ellipses represent multivariate normal distribution (CI = 0.95) for each included location. (d) a close-up of the first and second visit blood sample PCoA weighted UniFrac distance ordination plot (Figure 2c). V1: first visit, V2: second visit.

To characterize the bacterial DNA composition from various locations, we first evaluated alpha diversity using the Shannon entropy index and beta diversity using PCoA of weighted UniFrac distances (Figure 2). Comparing the alpha diversity indexes, no significant difference was observed between blood samples from both visits, but it was observed when comparing V1 blood samples to other locations (Figure 2a). As expected, oral and fecal samples displayed distinctly higher alpha diversity index than other locations. The median Shannon index of blood samples was 2.29 (IQR = 1). A graphical representation of the diversity of Pielou’s evenness and Faith’s PD indexes is shown in Fig. S2, S3 (Supplementary File 2).

PCoA analysis showed the grouping of the samples according to the collection site, with the most distinct grouping of fecal samples. Based on this analysis, blood samples overlapped with the samples from different skin locations. Beta diversity of blood samples alone depicted substantial similarity between the two blood samples from each individual (Figure 2b-d); weighted UniFrac ordination for all samples prior to decontamination is shown in Fig. S4 (Supplementary File S2). Boxplots representing weighted and unweighted UniFrac distance value distributions for each location are shown in Fig. S5, S6.

### Bacterial DNA composition in blood and other body sites

**Figure 3.**
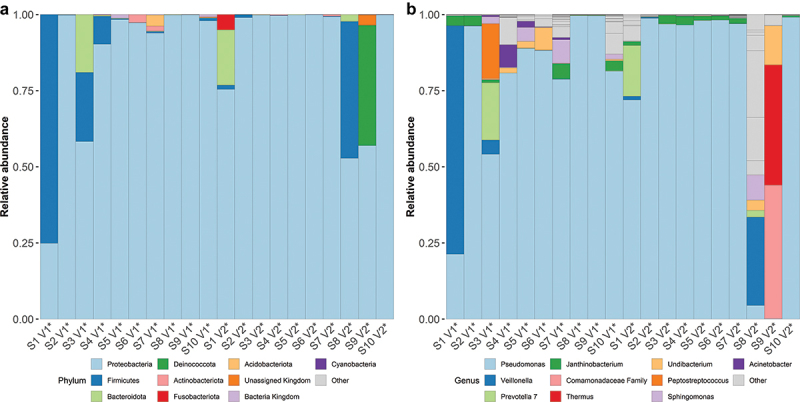
Taxa plot depicting relative abundances of most abundant taxa in all blood microbiome samples:(a) phylum level, (b) genus level. Each participant is assigned a unique identification code. V1: first visit, V2: second visit.

An overall characterization of the taxonomic profile in all collected blood samples is depicted in Figure 3. At the phylum level, Proteobacteria predominated in almost all V1 and V2 blood samples (91.8%), followed by Firmicutes (4.5%), and Bacteroidetes (1.7%) (Figure 3a). *Pseudomonas* was the most frequent genus (86.1%), while *Veillonella* (3.2%), *Prevotella_7* (1.6%), and others were less common (<1.5%) (Figure 3b).

Microbiome composition at the genus level for other locations is shown in Fig. S7 – S13 (Supplementary File S2). In summary, the most frequent genera in fecal samples were *Faecalibacterium*, *Bifidobacterium*, and *Bacteroides*; in oral samples, *Streptococcus* and *Haemophilus*; in vaginal samples, *Lactobacillus* and *Streptococcus*; and in skin samples, *Ralstonia* and *Staphylococcus*.

### Origin and stability of blood microbiome

To investigate the potential origin of bacterial DNA in blood, we analyzed the presence of exact ASVs found in V1 blood samples and compared them to samples collected simultaneously from other locations on the same individual (Table 1). We observed that 10.3–35.0% of the ASVs identified in blood were also found in samples from other body sites, with the highest overlap occurring in skin swabs. As a key finding in our study, we identified 32 ASVs uniquely shared only between blood and a single location within an individual, with no overlap across multiple locations in the same participant (Table 2). Importantly, such singleton ASV pairs were not observed when comparing samples across different individuals excluding the possibility that uniquely shared ASVs are the result of contamination. Other ASVs found to be identical in blood and other locations were identified in more than one participant (Table 2).

**Table 1.** Observed overlap of ASVs between V1 blood samples, other V1 samples, and V2 blood samples.

Participant	Number of ASVs in V1 blood samples	Number of ASVs both in V1 blood sample and at least one other location of the same individual (%)	The proportion of ASVs from V1 blood samples as a percentage of all ASVs observed in the individual (%)	Number of ASVs in V2 blood samples	Overlap between V1 and V2 blood samples (%)
S1	18	5 (27.78)	1.77	28	9 (24.32)
S2	20	5 (25.00)	2.36	25	12 (36.36)
S3	29	3 (10.34)	3.40	21	11 (28.21)
S4	19	6 (31.58)	2.31	23	3 (7.69)
S5	26	7 (26.92)	3.49	22	10 (26.32)
S6	18	6 (33.33)	1.81	22	11 (37.93)
S7	44	7 (15.91)	4.63	45	11 (14.10)
S8	20	7 (35.00)	3.14	18	2 (5.56)
S9	20	6 (30.00)	4.04	8	2 (7.69)
S10	40	9 (22.50)	6.49	23	14 (28.57)
***Median***	***20***	***6 (27.35)***	***3.27***	***22.5***	***10.5 (25.32)***
***IQR***	***9***	***1.75 (8.06)***	***1.58***	***3.25***	***6.5 (19.19)***

IQR: interquartile range, ASV: amplicon sequence variant. V1: first visit, V2: second visit after three months.

**Table 2.** Observed overlap between V1 blood samples and other V1 samples representing specific microbiome subpopulations.

Location	Number of ASVs concurrent with V1 blood samples (%)	Genera representing unique ASVs identical between blood and the particular location found in a single participant (number of different ASVs)	Genera representing ASVs identical between blood and the particular location found in more than one participant (number of different ASVs)
Oral mucosa	23 (0.53)	*Lawsonella (1); Rothia (1); Veillonella (1)*	*Lawsonella (1); Pseudomonas (3); Ralstonia (1); Rothia (1); Streptococcus (2); Veillonella (1)*
Scalp	31 (0.71)	*Acinetobacter (1); Brevundimonas (1); Micrococcus (1); Paracoccus (1); Streptococcus (1); Undibacterium (1)*	*Acinetobacter (1); Bifidobacterium (1); Brevundimonas (1); Lawsonella (2); Micrococcus (1); Paracoccus (1); Pseudomonas (3); Ralstonia (2); Staphylococcus (2); Streptococcus (1); Undibacterium (1)*
Upper back	36 (0.83)	*Acidovorax (1); Bifidobacterium (1); Lawsonella (1); Micrococcus (1); Paracoccus (1); Rothia (1); Sphingomonadaceae_Unclassified (1); Streptococcus (1); Undibacterium (1); Veillonella (1)*	*Undetermined (1); Acidovorax (1); Bifidobacterium (1); Brevundimonas (1); Lawsonella (1); Micrococcus (1); Paracoccus (1); Pseudomonas (4); Ralstonia (2); Rothia (1); Staphylococcus (3); Streptococcus (1); Undibacterium (1); Veillonella (1)*
Volar forearm	38 (0.88)	*Bifidobacterium (1); Chloroplast (1); Corynebacterium (1); Paracoccus (1); Rothia (1); Streptococcus (1); Sutterella (1); Veillonella (1)*	*Bifidobacterium (1); Brevundimonas (1); Chloroplast (1); Corynebacterium (1); Paracoccus (1); Pseudomonas (2); Ralstonia (2); Rothia (1); Staphylococcus (3); Streptococcus (1); Sutterella (1); Undibacterium (1); Veillonella (1)*
Vagina	19 (0.44)	*Prevotella_7 (1); Staphylococcus (1)*	*Pseudomonas (4); Ralstonia (1); Staphylococcus (1); Undibacterium (1)*
V1 feces	22 (0.51)	*Bacteroides (1); Blautia (1); Sutterella (1)*	*Bacteroides (1); Bifidobacterium (2); Blautia (1); Pseudomonas (2); Ralstonia (1); Sutterella (1)*

IQR: interquartile range, ASV: amplicon sequence variant, V1: first visit.

To evaluate the similarity of the bacterial DNA composition in blood, we used LEfSe to compare samples collected during the first visit with samples collected after three months. We found no statistically significant differential abundance results for ASVs or other taxonomic ranks when comparing blood and fecal samples between the first and second visits. Observed ASVs partially overlapped between the longitudinally collected whole blood samples (Table 1).

## Discussion

There is a growing body of evidence in the scientific literature suggesting the presence of bacteria in the human blood, not only in septic patients but also in healthy individuals. There are various hypotheses about the potential origin of the blood microbiome, but it has not been evaluated in detail. This study determined the presence of bacterial DNA in various samples from the same individual by analyzing the 16S rRNA gene amplicons.

The key finding in our study is 32 unique ASVs that were identical between the blood and a single location within an individual, with no overlap across multiple locations in the same participant. Using ASVs to compare the presence of the same bacteria in different samples provides a much better resolution to track down the exact source of bacterial presence. We, therefore, specifically searched for 100% identical ASVs between blood and other body locations to increase accuracy.

It is not surprising that overall the large number of sequence variants identified as identical in a blood sample and other locations were present in several individuals, as they represented commonly found bacterial taxa. On the other hand, one may not exclude contamination in such cases even after applying stringent measures to filter out the possible contaminants, especially considering that studies use different decontamination methods that may affect the true results.^30,54,55^

It is also important to note that the decontamination strategy used in this study was adapted to account for the low-biomass samples. While this approach may differ from those used for high-biomass samples, our filtering method aims to reduce bias and improve reproducibility in microbial detection from low-biomass samples.

While contamination remains a potential concern in low-biomass DNA sequencing studies, the identification of 32 unique ASVs that overlap only within individual participants significantly reduces the likelihood that these sequences are contaminants. Additionally, the absence of cross-individual ASV overlap acts as strong evidence against cross-contamination.

Our finding suggests that bacterial DNA detected in blood may originate from localized sites, such as the skin, and sporadically transfer into the bloodstream as bacteria or bacterial DNA. Our study is the first providing observational data that supports the idea that such sporadic transfer from other locations to the blood is the most possible origin of bacterial DNA in the blood.

In samples with low and very low microbial biomass, contamination can account for up to 90% of the resulting reads; therefore, the inclusion of negative controls at different stages of sample processing and their analysis is very critical.^25^ Due to the relatively recent focus on the importance of negative controls in studies with low microbial biomass samples, many studies lack appropriate negative and blank controls subjected to sequencing. This problem has generally affected microbial research, giving a false view of the microbiome composition of different low-biomass samples.^16^ For instance, previous studies have shown that the placenta has its unique microbiome.^1,6^ Olomu et al., on the contrary, have shown that after correcting for *kitome* and cross-contamination events, it was not possible to identify a placenta-specific microbiome.^37^ This aspect has been reviewed by analyzing the latest studies on this subject and stressing pitfalls in the research of low-biomass samples.^26^ Therefore, we paid particular attention to the analysis of negative controls, including a number of negative controls from different working areas and reagents.

Previous studies analyzing the content of blank controls have identified both human microbiome-associated taxa, such as *Prevotella* and *Lactobacillus*, and members of the genus *Janthinobacterium* and *Thermus*, commonly found in water and hot springs typically not present in human-associated microbiomes.^12,38^ Most commonly, bacteria of the genus *Ralstonia* are identified in the blank controls. These bacteria are found in plants, soil, and water and are typical laboratory contaminants.^16,47,48,60^ On the other hand, this genus cannot be entirely excluded from data since *Ralstonia* is often associated with human opportunistic pathogenic microbiota, especially in hospital settings.^2,5,47^ Furthermore, previous reports have found that members of the genus *Ralstonia* were associated with intestinal diseases,^27^ cystic fibrosis,^22^ and bladder cancer.^9^ In other studies, various reagent kits were tested and analyzed, resulting in the identification of the *Pseudomonas* genus accounting for most of the contamination (>70%), followed by the *Burkholderia* genus (17%),^7^ while Whittle et al. observed *Serratia* as a contaminant in the blank controls of blood microbiome study^60^ and Gosiewski et. al. – *Sphingomonas*.^21^ However, in many samples, the majority of taxonomic composition was formed by bacteria widespread in the environment, and it can therefore be part of the human microbiome in the locations that frequently interact with this environment.

The presence of nucleic acids from Gram-negative bacteria was observed in the blood, with a strong dominance of Proteobacteria phylum, *Pseudomonas* genus, followed by Firmicutes and Bacteroidetes, similar to what has been found in other studies.^39,44,60^ On the contrary, T. Gosiewski et al. observed a significant predominance of anaerobic bacteria, including Gram-positive Bifidobacteriales order, and decreased abundance of Proteobacteria phyla in healthy volunteers compared to patients with sepsis,^21^ which partly coincides with Szabó et al. study where the abundances of *Enhydrobacter* and *Pseudomonas* genera of Proteobacteria phyla and *Micrococcus* genus of Actinobacteria phylum were statistically significantly higher in the sepsis group compared to control group.^54^ There are controversial observations in the literature about the *Pseudomonas* genus as it is ubiquitous in the environment, including humans, animals, and plants.^40^ It cannot be excluded that their presence in the blood samples may result from potential contamination^59,60^ as the *Pseudomonas* were present in negative controls. On the other hand, due to their overwhelming presence in the environment, bacteria of this genus may predominate in very low biomass samples naturally, as this trend has been observed in other studies of the lung microbiome,^17^ the deeper layers of the dermis,^35^ and the fetal tissues,^34^ which were previously considered sterile. Several studies have also used the classical culture-based method to determine the presence and composition of a blood microbiota. Panaiotov et al. found that in healthy people, the microorganisms in the blood are in an inactive state, while under appropriate conditions, they may change their activity status, also confirming that the predominant phylum of bacteria in the blood is Proteobacteria.^41^ Whittle and colleagues identified the *Staphylococcus* genus by culturing blood samples, which more likely was the result of contamination from skin,^60^ meanwhile recently Tsafarova et al. confirmed the possible existence of blood microbiota using light and transmission electron microscopy as well as culturing freshly drawn whole blood, where they observed living bacterial cells undergoing complex life cycles in peripheral blood mononuclear cells.^57^ Taking into account that 16S rRNA sequencing is a low-resolution method, we compared our results with metagenomics studies as well. In An et al. study comparing the blood microbiome of healthy controls versus breast cancer patients, *Pseudomonas* was the dominant genus in the control group, followed by *Streptococcus*, *Lactobacillus*, *Staphylococcus*, *Acinetobacter*, *Enhydrobacter*, *Corynebacterium 1*, *Cutibacterium*, *Sphingomonas*, and *Cupriavidus*.^3^

Most blood samples showed a strong dominance of the *Pseudomonas* genus, but sequences of other microorganisms were also detected. Although in much smaller numbers, we also found *Streptococcus, Staphylococcus, Lactobacillus, Veillonella*, and other bacterial genera, - resembling the above-mentioned composition. These are typical members of oral, gut, vaginal, and skin microbiomes and were found in different proportions in all these locations, while *Brevundimonas, Sphingomonadaceae_Unclassified* were only found in all skin locations. It is considered that microorganisms enter the circulation from different body parts, such as the gut.^39^ This has been confirmed in the case of various gastrointestinal and other diseases in which intestinal permeability has been altered.^52,53,56^ At the same time, Shah et al. later found that the blood microbiome does not directly reflect the gut microbiome.^53^ The same notions are supported by a mice study where changes in gut microbiome did not reflect on blood microbiome composition suggesting that these two environments are not as closely related regarding microbiome as previously thought.^29^ Our study confirms this observation as the beta diversity analysis shows that there is a much larger distance between blood and fecal samples than between blood and skin samples, suggesting that the microbial signatures in the blood of healthy individuals may originate from the skin microbiome, which partly coincides with Whittle et al. results.^60^ In the same study, it was observed that the blood microbiome is also similar to the oral microbiome.^60^ In our study, bacterial taxa from blood mostly overlap with those from various other skin samples. Still, a significant fraction of bacteria found in the blood did not overlap with other locations. This may suggest that the blood has its own unique microbiome maintained independently for a longer time. However, it should be noted that due to practical reasons, some locations were not tested (e.g., respiratory tract), and also, the skin microbiome may contain a significantly larger number of different bacteria, as only a tiny fraction of the whole skin is tested even if several different locations were chosen.

Paise et al. examined different fractions of blood, considering previous studies that suggested some bacteria may be dormant in red blood cells. They found significant differences between the taxonomic profiles of the different blood fractions, where potential blood pathogens that we also found, such as *Acinetobacter*, *Corynebacterium*, *Pseudomonas*, *Staphylococcus*, were relatively more abundant in the red blood cell fraction.^39^ Therefore, whole blood samples were used in this study. Moreover, a feasibility study concluded that whole blood samples could provide a broader picture than individual blood fractions while also reducing the risk of microbial contamination due to the smaller number of laboratory interventions (*unpublished data*). In addition, since blood is considered to have a particularly low level of microbial biomass and thus is more sensitive to contamination, a different DNA extraction protocol was used for blood compared to the other samples. To extract the maximum amount of microbial DNA, whole blood from EDTA tubes was treated with the phenol/chloroform extraction method.

Interestingly, non-human-associated taxa were also observed in the blood in this study. The *Chloroplast* sequences were found on the forearm and in the blood sample of one individual. Most likely, this genus, usually associated with plants and algae,^20^ entered the bloodstream from a forearm injury. Another study has found the presence of a non-human microbiome genus in individuals regularly using herbal cosmetics.^7^ In some studies, OTUs and bacteria classified as mitochondria, chloroplasts, or eukaryotes are filtered out from the outset.^32^ Almost all chloroplast DNA identified in our study was present only in skin samples.

Alpha diversity of taxa in blood samples is relatively low, with a median Shannon index of 2.14, a value that is similar to the findings of other studies.^44^ An equal index value was also calculated for skin samples, while it was 3 times higher for fecal samples.

To determine the compositional stability of the found microbial DNA over time, blood and fecal samples were collected twice at 3-month intervals. The linear discriminant analysis (LDA) effect size (LEfSe) algorithm did not show significant differences in either the ASV or genus level data, indicating that the blood and gut microbiome of these individuals remained stable during the three months. The major bacterial taxa found in the blood overlapped between the samples from both visits, suggesting that the overall blood microbiome remains stable in healthy subjects.

Microbiome stability has also been confirmed by other studies, as the composition of the intestinal and the skin microbiomes in healthy individuals can persist for years,^19,36^ with certain fluctuations influenced by various factors over time.^13^ Our results overall support recent findings of^55^ indicating that blood does not have its core microbiome and microbes enter in this environment sporadically. At the same time, our study shows that this pattern is consistent, resulting in a similar microbiome composition in samples taken several months apart.^55^ However, we acknowledge that the observed similarity in bacterial DNA profiles over time does not necessarily imply a persistent endogenous blood microbiome and may instead reflect ongoing or intermittent microbial shedding from other body sites.

Some limitations of our study should be mentioned. First, we had a relatively small sample size and rather a heterogeneous study group with respect to age, sex, and BMI. However, our primary goal was not to associate specific blood microbiome composition with phenotype but to evaluate the origin of the blood microbiome compared with the microbiome from other locations. For this purpose, having a phenotypically heterogeneous group may be seen as an advantage. Secondly, different methods were used to isolate microbial DNA from blood samples and swabs, and the DNA extracted from blood samples contained a large amount of human DNA. Therefore, we filtered out those ASVs that were present in greater numbers in control samples than in biological samples, according to Karstens et al. paper,^25^ while others have excluded overlapping sequences between the samples and controls.^41^ We chose this method so that the taxa that are characteristic of the human microbiome would not be filtered out, but as a result, the data used in the analysis include some taxa that are usually not associated with the human microbiome. We also did not estimate the viability of bacterial cells. It is therefore impossible to determine whether the genomic sequences detected in the blood originate from live bacteria or whether only their genetic material is present in the circulation. For this reason, we took a cautious approach in interpreting the results.

## Conclusions

To the best of our knowledge, this is the first study presenting results on origin of the microbial DNA in blood by analyzing samples collected from multiple locations from the same individuals at the same time point and tracing bacterial sequences with a qualitative approach. Our results confirm the sporadic nature of blood microbiome predominantly originated by horizontal transfer from other locations. Despite the small sample size, this study depicts results that add new knowledge on the blood microbiome that can be further used in designing and interpreting other studies.

## Supplementary Material

Supplementary_File_2.DOCX

Supplementary_File_1.xlsx

## Data Availability

The dataset supporting the conclusions of this article is available in the Sequence Read Archive repository under the accession project number PRJNA874911 (https://www.ncbi.nlm.nih.gov/bioproject/874911).

## Abbreviations

16S rRNA gene: 16S ribosomal RNA gene
ASV: Amplicon sequence variant
BMI: Body mass index
IQR: Interquartile range
PCR: Polymerase chain reaction
V1: First visit
V2: Second visit after three months
