# Supplementary material for: Tracking the origin of bacterial DNA in blood: Indication of localized and sporadic transfer from other body sites
Source: Gut Microbes Rep. 2025 Apr 1;2(1):2482771. doi: 10.1080/29933935.2025.2482771 (PMC12940155; doi:10.1080/29933935.2025.2482771)
Supplement: Supplementary_File_2.DOCX [file KGMR_A_2482771_SM1525.docx]

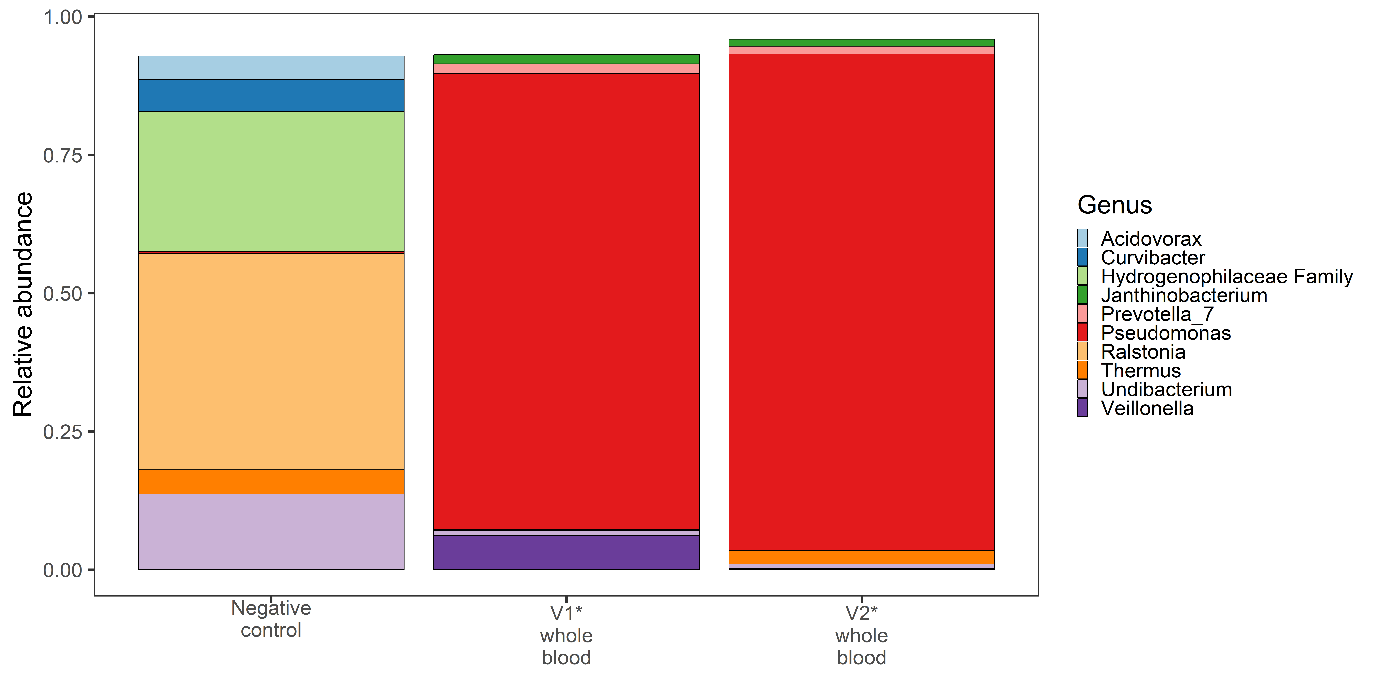


Figure S1. Microbiome composition in negative controls and blood samples from both visits at the genus level. V1: first visit, V2: second visit.


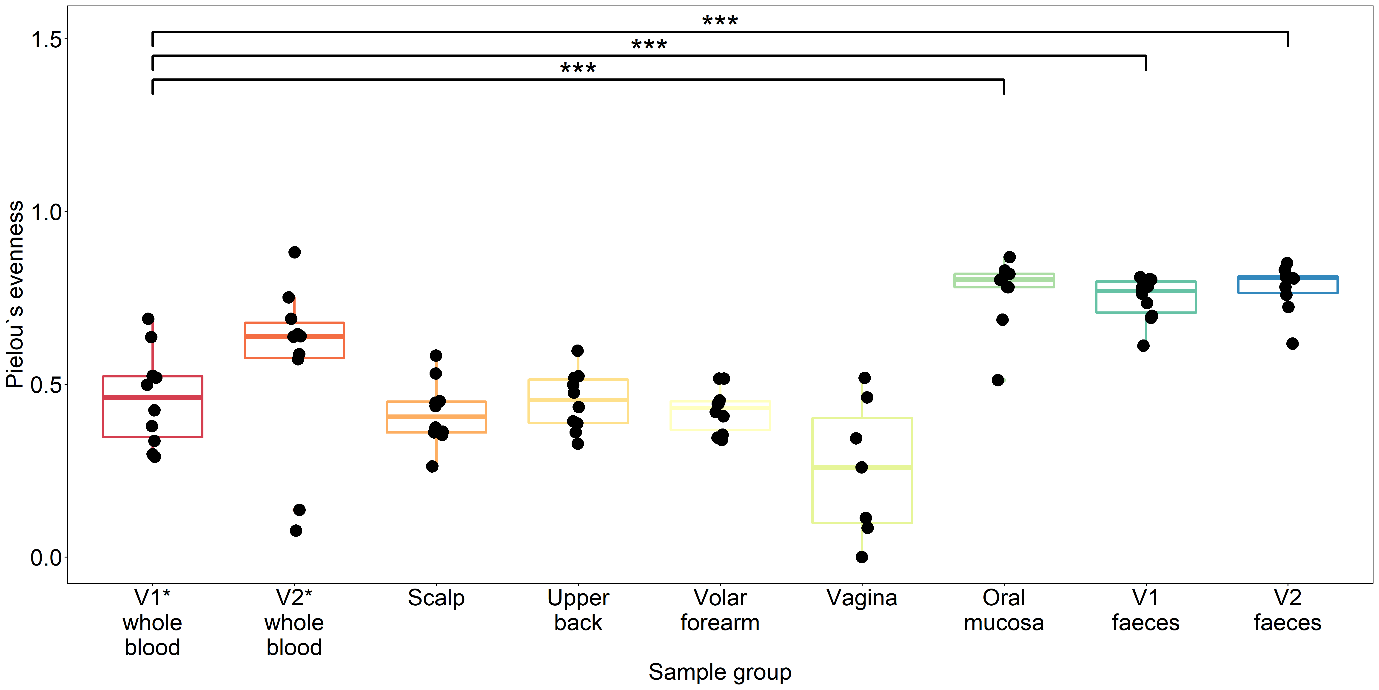
Figure S2. Graphical representation of the alpha diversity of Pielou's evenness index. V1: first visit, V2: second visit.


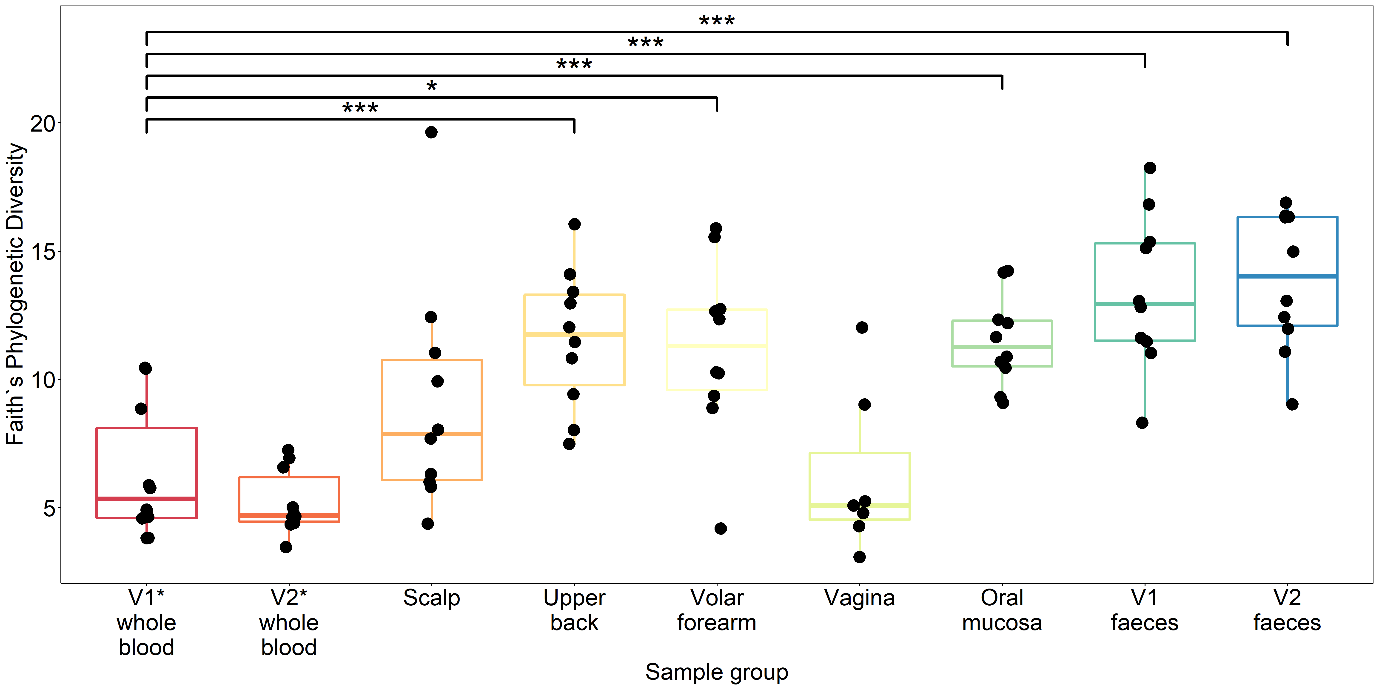
Figure S3. Graphical representation of the alpha diversity of the Faith’s PD index. V1: first visit, V2: second visit.


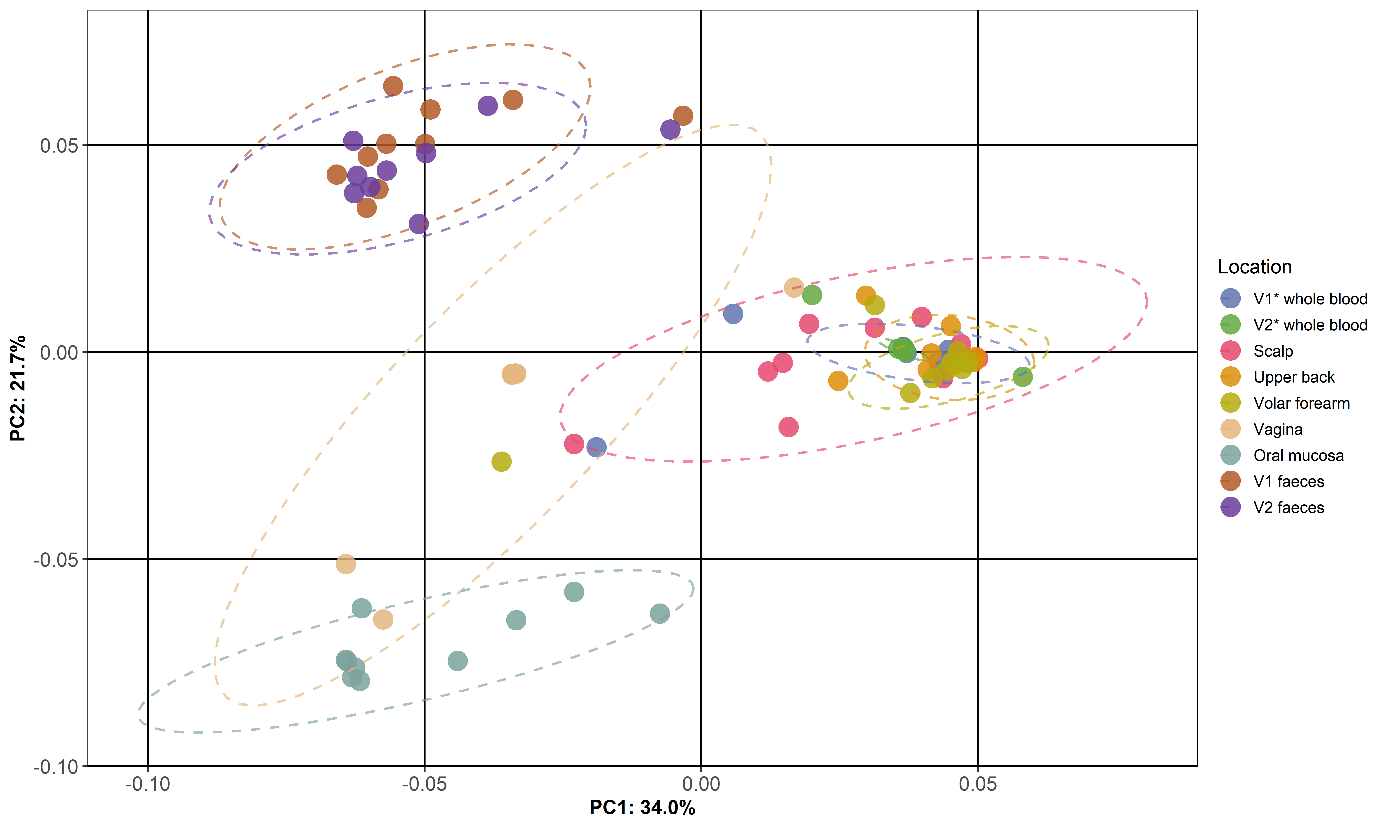


Figure S4. Representation of beta diversity for all true samples with PCoA weighted UniFrac distance ordination plot before decontamination procedure.


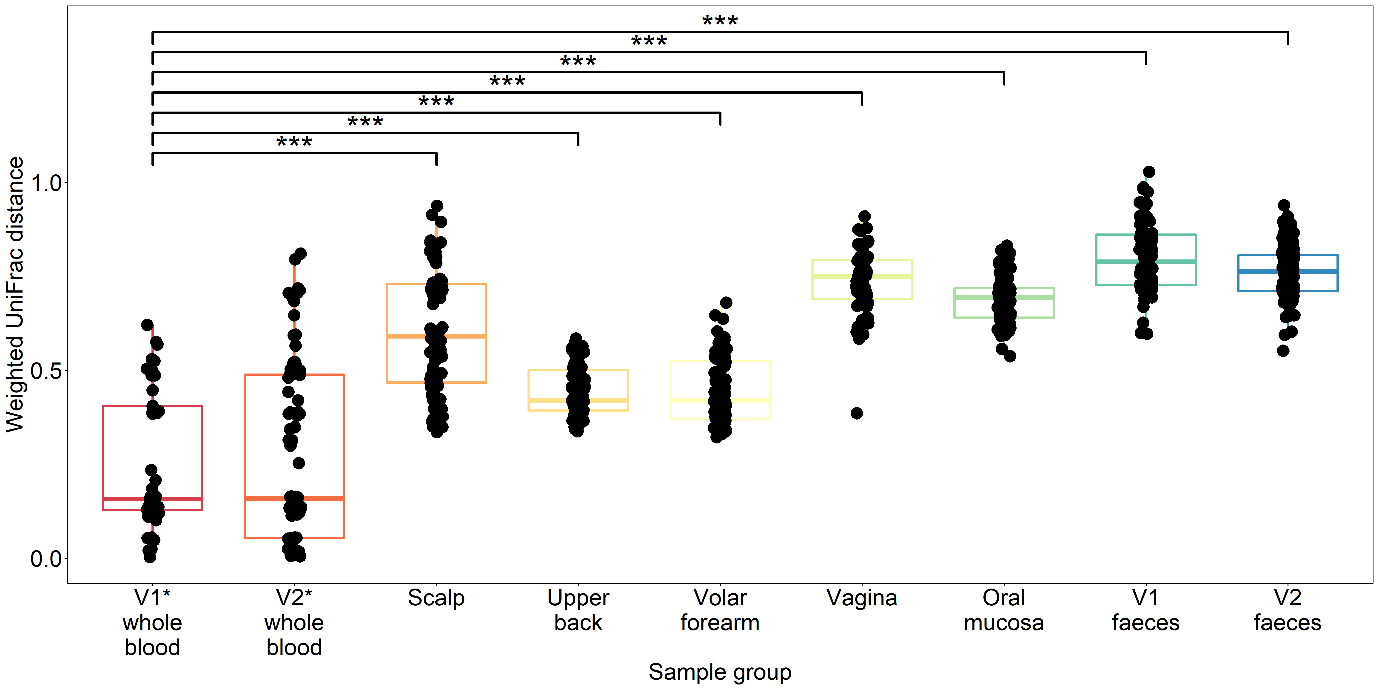
Figure S5. Boxplots representing weighted UniFrac distance value distributions for each location. V1: first visit, V2: second visit.


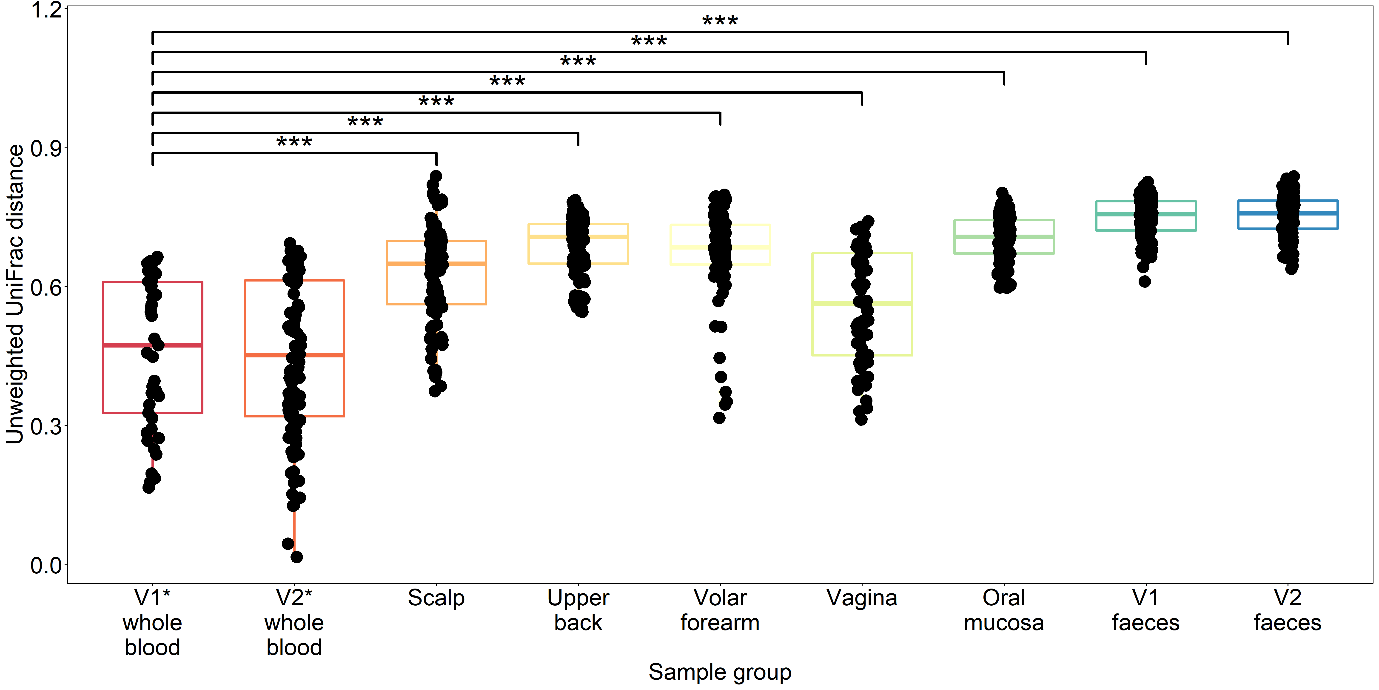
Figure S6. Boxplots representing unweighted UniFrac distance value distributions for each location. V1: first visit, V2: second visit.


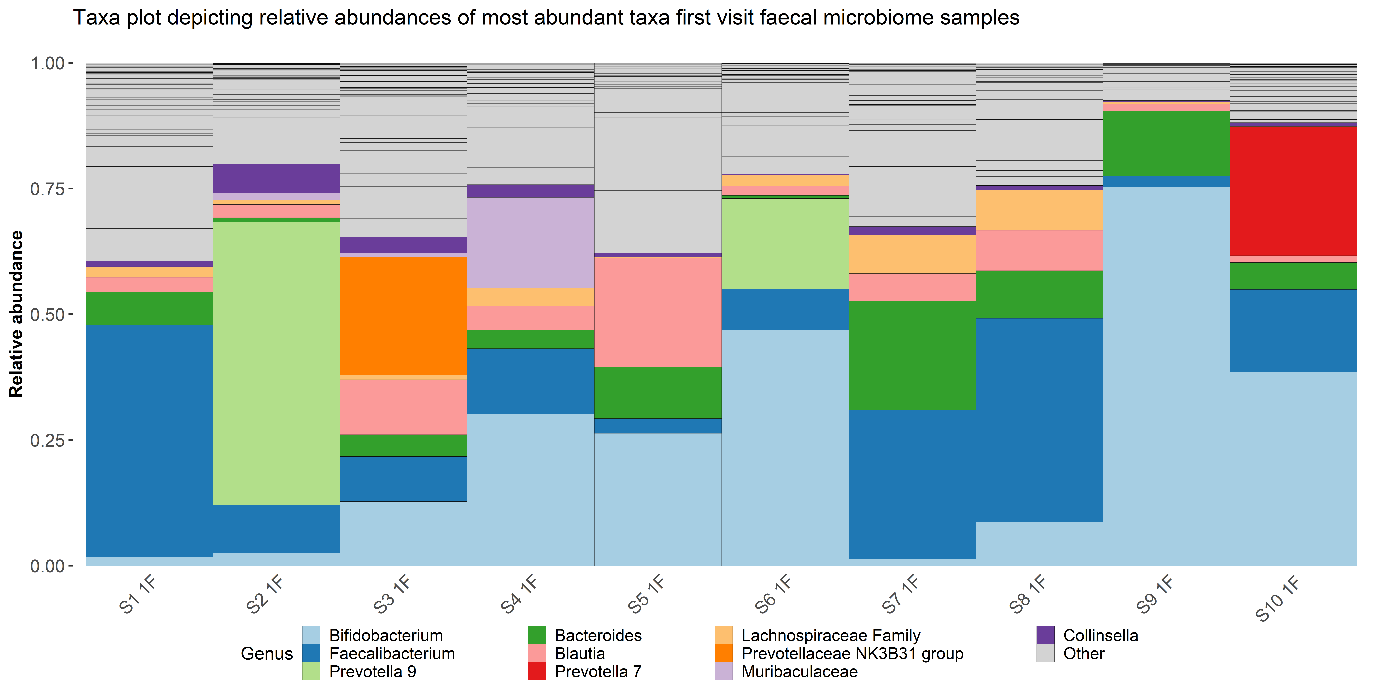
Figure S7. Microbiome composition in first visit faecal samples (1F) at the genus level.


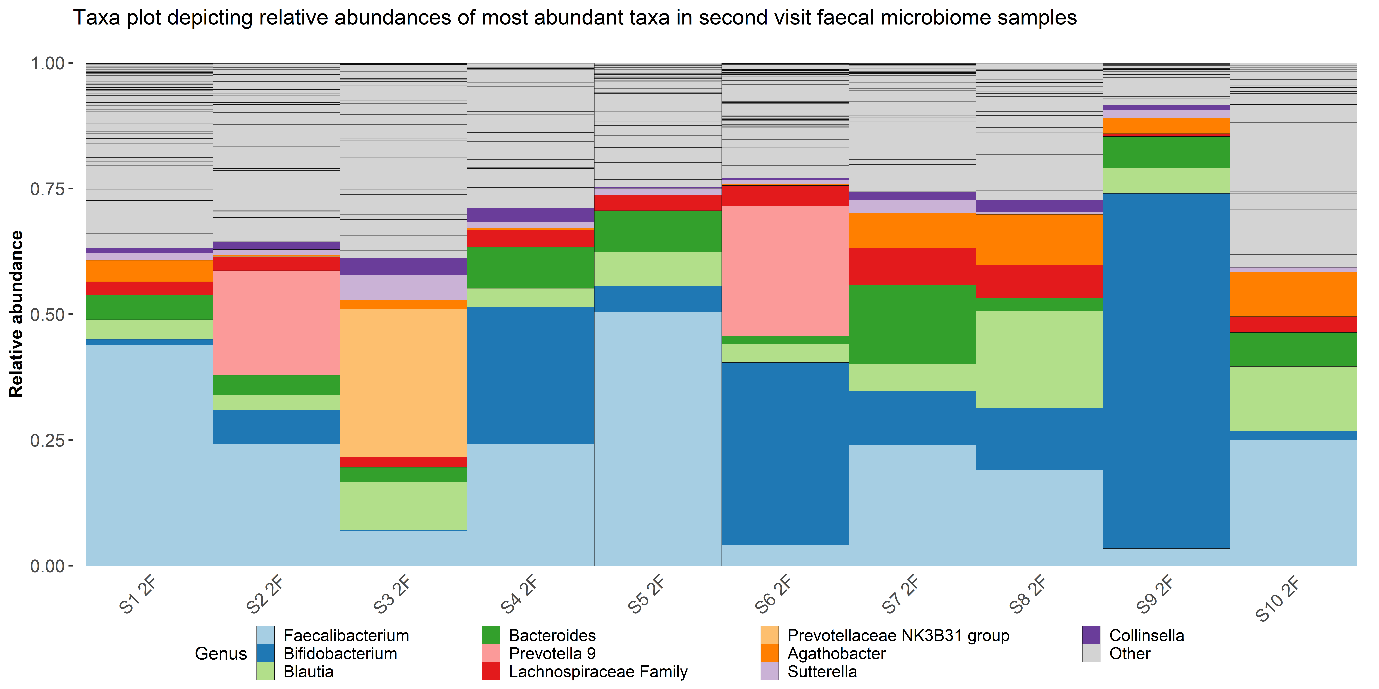
Figure S8. Microbiome composition in second visit faecal samples (2F) at the genus level.


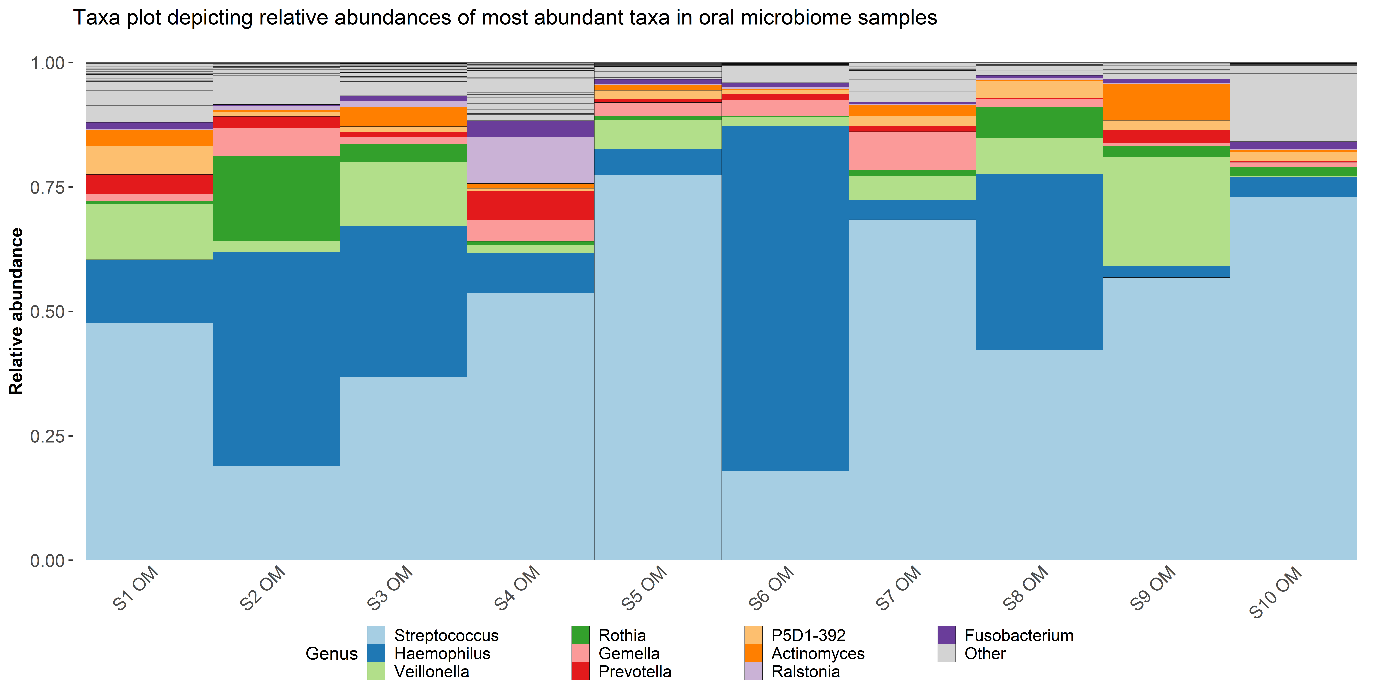
Figure S9. Microbiome composition in oral mucosa samples (OM) at the genus level.


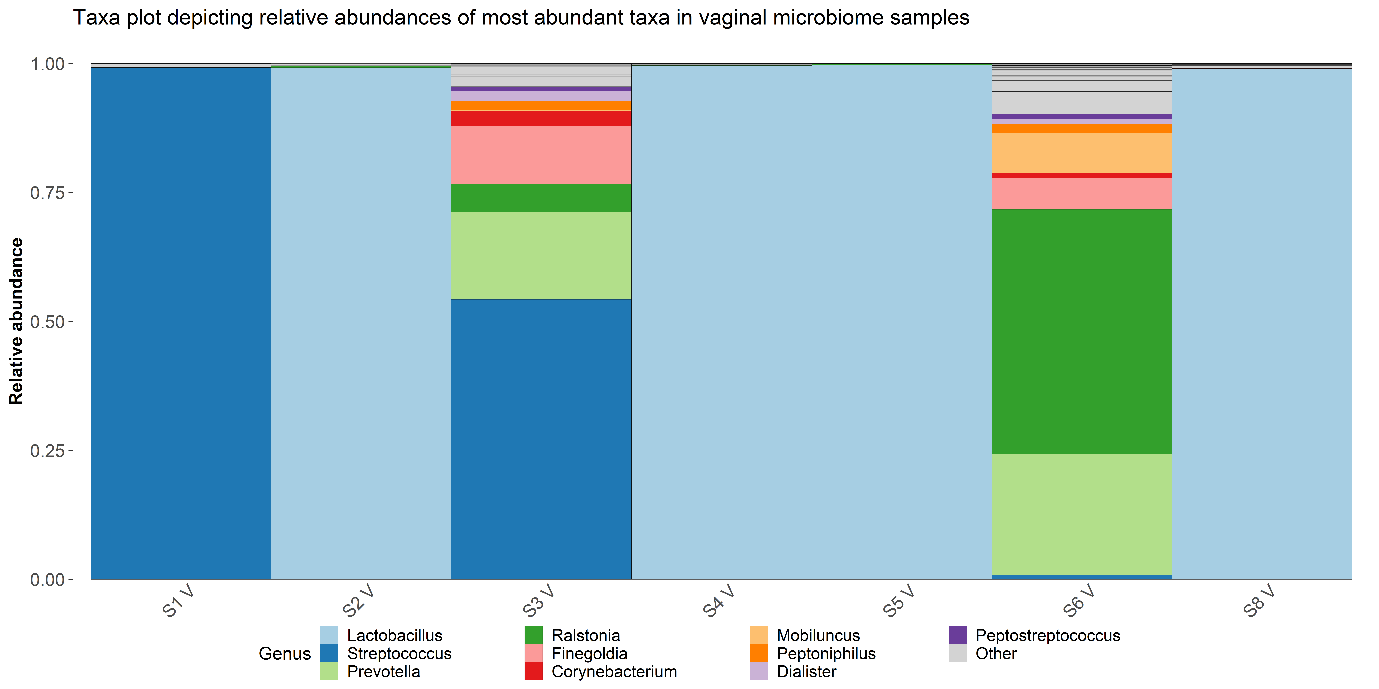
Figure S10. Microbiome composition in vaginal samples (V) at the genus level.


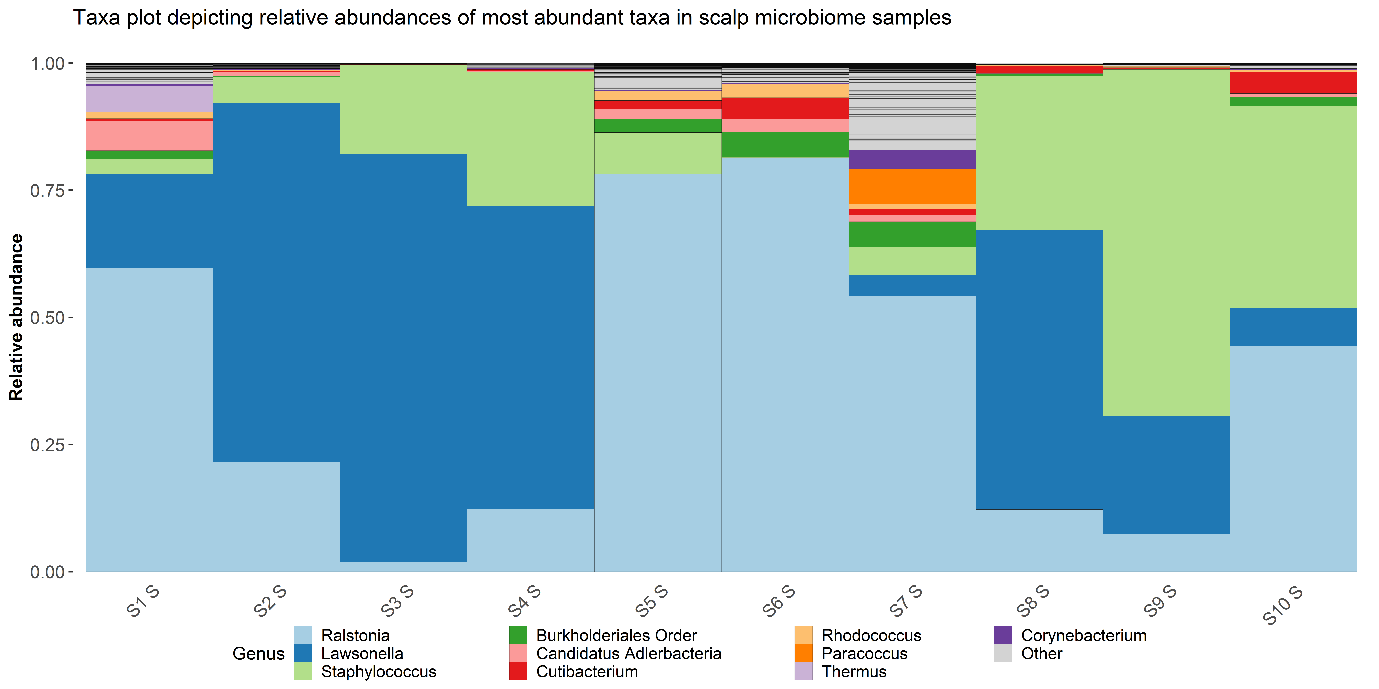
Figure S11. Microbiome composition in scalp samples (S) at the genus level.


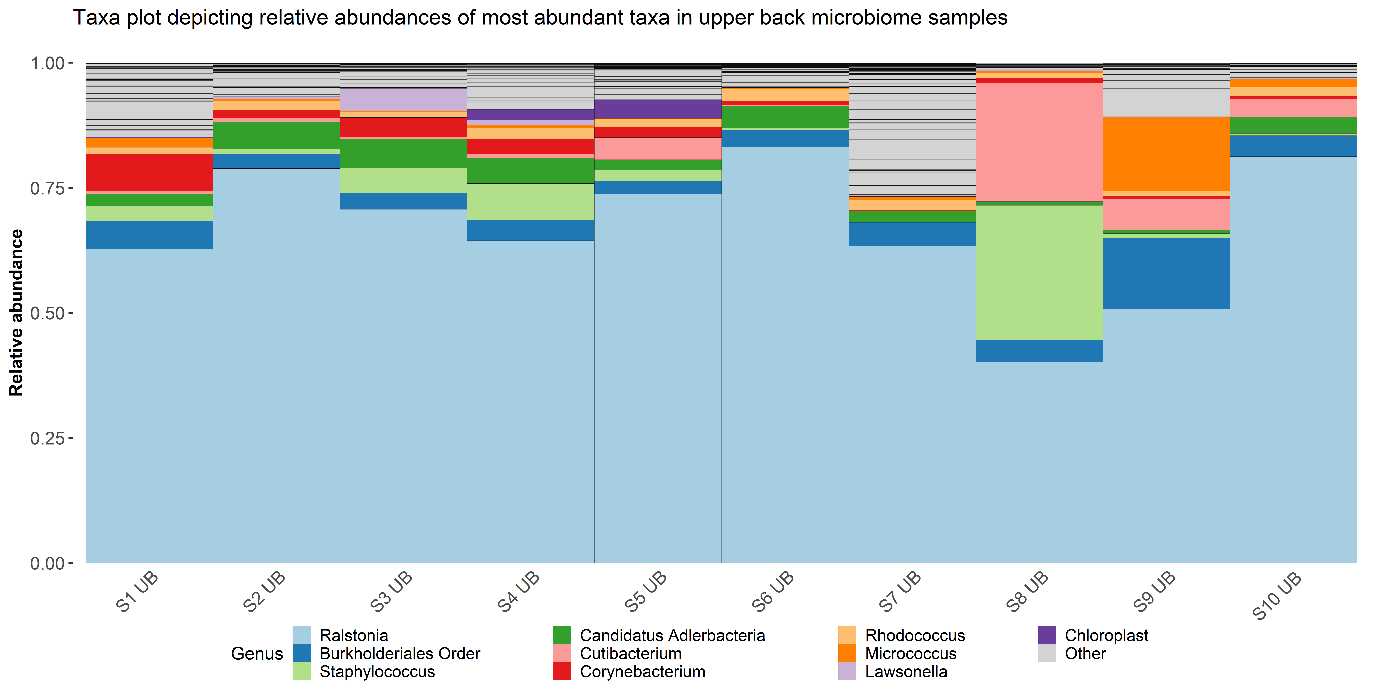
Figure S12. Microbiome composition in upper back samples (UB) at the genus level.


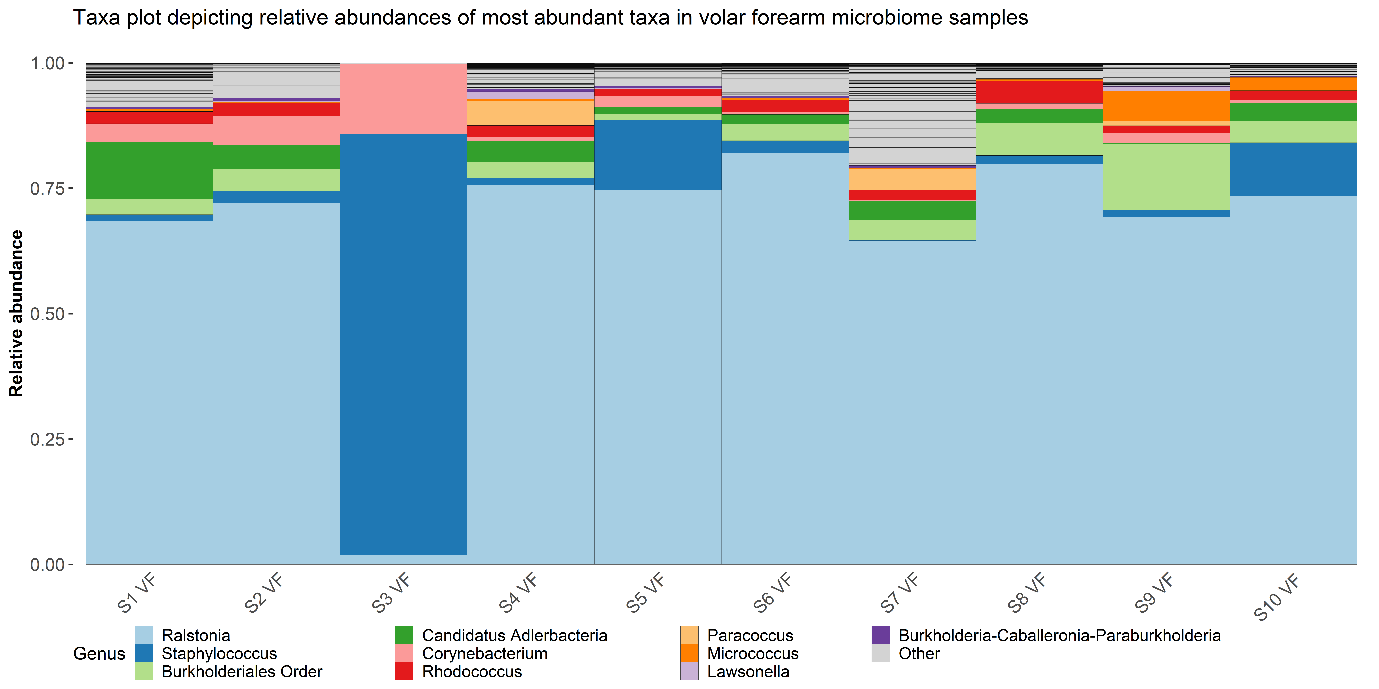
Figure S13. Microbiome composition in volar forearm samples (VF) at the genus level.
